# Photocatalytic Antibacterial Performance of Glass Fibers Thin Film Coated with N-Doped **SnO**_**2**_ **/TiO**_**2**_

**DOI:** 10.1155/2014/869706

**Published:** 2014-02-12

**Authors:** Peerawas Kongsong, Lek Sikong, Sutham Niyomwas, Vishnu Rachpech

**Affiliations:** ^1^Department of Mining and Materials Engineering, Faculty of Engineering, Prince of Songkla University, Hat Yai, Songkhla 90112, Thailand; ^2^Department of Mechanical Engineering, Faculty of Engineering, Prince of Songkla University, Hat Yai, Songkhla 90112, Thailand

## Abstract

Both N-doped and undoped thin films of 3SnO_2_/TiO_2_ composite were prepared, by sol-gel and dip-coating methods, and then calcined at 600°C for 2 hours. The films were characterized by FTIR, XRD, UV-Vis, SEM, and XPS, and their photocatalytic activities to degrade methylene blue in solution were determined, expecting these activities to correlate with the inactivation of bacteria, which was confirmed. The doped and undoped films were tested for activities against Gram-negative *Escherichia coli* (*E. coli*) and *Salmonella typhi* (*S. typhi*), and Gram-positive *Staphylococcus aureus* (*S. aureus*). The effects of doping on these composite films included reduced energy band gap, high crystallinity of anatase phase, and small crystallite size as well as increased photocatalytic activity and water disinfection efficiency.

## 1. Introduction

The supply of safe drinking water is an issue of concern, particularly in developing countries. Although several initiatives have been successful in supplying safe drinking water to urban populations, the extent of these efforts still falls short of the required targets for sustainable development. In developing countries, water delivery systems are plagued by leakages, illegal connections, and vandalism, and precious water resources are mismanaged. In Africa, Asia, Latin America, and the Caribbean, nearly one billion people in rural areas have no access to sufficient clean water supplies [1]. Contaminated water commonly contains dangerous pathogens, and its consumption creates serious health effects and societal problems. In the past decade, many innovative disinfection technologies were developed and adopted as alternatives to chlorine and ozone associated disinfection processes, including germicide ultraviolet (UV) radiation and photocatalytic oxidation. The traditional disinfection approaches have potential risks, such as carcinogenic byproducts (DBPs). For the alternative technologies, diverse nanophotocatalysts such as titanium dioxide (TiO_2_), zinc oxide (ZnO), cadmium sulfide (CdS), and silver nanoparticles have been widely studied and are considered promising due to their unique properties including large specific area and high reactivity [2].

TiO_2_ is the most commonly used semiconductor photocatalyst and the most studied of the various nanomaterials. Activated by UV-A irradiation, its photocatalytic properties have been utilized in various environmental applications to remove contaminants from both water and air. A wealth of information on TiO_2_ photocatalytic inactivation of bacteria has been acquired over the last 20 years. TiO_2_ can kill both Gram-negative and Gram-positive bacteria, although Gram-positive bacteria are able to form spores and therefore are less sensitive. The exact bactericidal mechanism of reactive oxygen species (ROS) is not yet fully known, but the photocatalytic activity of TiO_2_ produces them, and they are extremely reactive killing or deactivating microorganisms on contact [3].

There are many techniques to improve photoactivity such as control of phase morphology, crystallite size, and reducing band gap energy. Doping TiO_2_ with N and combining it with SnO_2_ could improve the photochemical activity [4, 5].

The aim of this work was to investigate the water disinfection efficiency of N-doped and undoped 3SnO_2_/TiO_2_ composite films under UV radiation. The quantity of dopants in TiO_2_ films was varied. The N-doped and undoped 3SnO_2_/TiO_2_ composite films were formed as coatings on glass fibers, and the photocatalytic antibacterial effects of these films against Gram-negative *Escherichia coli* (*E. coli*), *Salmonella typhi* (*S*. *typhi*), and Gram-positive *Staphylococcus aureus* (*S. aureus*) were assessed. The fraction of viable bacteria that survived the treatment was determined with the spread plate technique. Furthermore, photocatalytic degradation of methylene blue dye in solution was also investigated, to correlate this activity with antibacterial activity.

## 2. Experimental

### 2.1. Materials and Methods

The TiO_2_ composite films were formed on glass fibers (E-type) with two coating layers. The specific surface area of the starting glass fiber materials is 2 m^2^ g^−1^ [6]. The first layer was 5SiO_2_/TiO_2_, and this film was prepared by adding titanium (IV) isopropoxide (TTIP, 99.95%, Fluka Sigma-Aldrich) dropwise under vigorous stirring into a mixture solution containing ethanol (99.9%, Merck, Germany) and tetraethylorthosilicate (TEOS, 98%, Fluka Sigma-Aldrich). The second coating layer was (optionally N-doped) 3SnO_2_/TiO_2_ composite. The N-doped 3SnO_2_/TiO_2_ was prepared by mixing 10 mL glacial acetic, 0.289 g ammonium carbonate, and 0.315 g Tin (IV) chloride pentahydrate. For the first coating layer the concentration of SiO_2_ was fixed at 5 mol%, while SnO_2_ doped in the second layer was fixed at 3 mol%. Nitrogen at 20 mol% was used for doping of the 3SnO_2_/TiO_2_ composite films, following Qin and coworkers [7]. The N-doped 3SnO_2_/TiO_2_ solutions were stirred at room temperature for 60 min, and then 2 M HCl was added into the solution to adjust its pH to about 3.5.

The glass fibers were first kept at 500°C for 1 h in order to remove wax and then carefully cleaned with ethanol. A dip-coating apparatus was used to coat the fibers. The first coating with SiO_2_/TiO_2_ acted as a buffer layer on the glass fibers, and N-doped 3SnO_2_/TiO_2_ sol was coated on the buffer layer as the second coating. A dipping speed of 1.0 mm/s into the sols gave homogeneous coatings. The coating films were turned into gels by drying at 60°C for 30 min. Then coated fibers were heated to 600°C at a heating rate of 10°C/min and held for 2 h. The coated glass fibers were cleaned by immersion in an ultrasonic bath of distilled water for 15 min in order to remove excess TiO_2_ particles. The TiO_2_ composite film coated glass fibers were dried at 105°C for 24 h. The samples were tested immediately after they had cooled in a desiccator to ambient temperature.

### 2.2. Material Characterization

Surface morphology was investigated by scanning electron microscopy (SEM) and energy-dispersive X-ray spectroscopy (EDS). XPS spectra were recorded with an AXIS Ultra DLD (Kratos Analytical Ltd., UK). Phase composition was characterized with an X-ray diffractometer (XRD) (Phillips E'pert MPD, Cu-K*α*). The crystallite sizes were estimated from XRD peaks using the Scherrer equation [8]:

(1) $$\begin{array}{c} D=\frac{0.9\lambda }{\beta \cos\theta_{\beta }}, \end{array}$$

where *D* is crystallite size, *λ* is the wavelength of X-ray radiation (Cu-K*α* = 0.15406 nm), *β* is the angle width at half maximum height, and *θ*_*β*_ in degrees is the half diffraction angle of the peak centroid. The FTIR transmittance spectra of the samples were also analyzed in order to confirm hydroxyl functional groups (TiO_2_–OH bonds) of the films. The band gap energies of TiO_2_ and TiO_2_ composites, in powder form, were measured by UV-Vis-NIR spectrometer with an integrating sphere attachment (Shimadzu ISR-3100 spectrophotometer) by using BaSO_4_ as reference. The onset absorbances were determined by the linear extrapolation of the steep part of the UV absorption toward the base line.

### 2.3. Photocatalytic Reaction Test

The photocatalytic activities of TiO_2_ and of N-doped 3SnO_2_/TiO_2_ thin films on glass fibers were tested by observing the degradation of methylene blue (MB). The MB solution (50 mL) had 1 × 10^−5^ M initial concentration, and 1 g [8] of undoped or doped TiO_2_ coated glass fibers were provided excitation from a 50 W UV-lamp (black light) in the 310–400 nm wavelength range, set at 32 cm distance from the samples. The photocatalytic reaction tests were done in a dark chamber, with various UV irradiation times up to 4 h. The remaining concentration of methylene blue was determined by UV-VIS spectrophotometer.

### 2.4. Photocatalytic Antibacterial Measurements

Gram-negative (*E. coli* and *S*.* typhi*) and Gram-positive (*S. aureus*) bacteria were obtained from the Microbiology Science Laboratory, Prince of Songkla University, Songkhla. The bacteria were grown aerobically in 4 mL of trypticase soy broth, at 37°C for 24 h. Then the bacterial solution was diluted in saline solution (0.85% NaCl) until the count of bacteria per milliliter of solution was in the range of 30–300. These counts were estimated by colony counter. The number of viable bacteria in a treated solution is readily quantified by spread plate technique, in which the sample is appropriately diluted and transferred to an agar plate. The grown colonies are counted, and each colony represents an initial viable bacterium in the plate culture. It is known that the initial bacterial concentration is an important factor affecting apparent antibacterial efficiency [9]. The initial bacterial concentration was set to about 10^3^ CFU/mL. A 50 mL aliquot of bacterial suspension was treated with a 40 g/L dose of coated glass fibers, with exposure to UV irradiation for various durations. Then, 1 mL of treated suspension was sampled and cultured on MacConkey agar plates (*E. coli* and *S. typhi*) or Nutrient agar plates (*S. aureus*), by incubation at 37°C for 24 h. After incubation, the colony counts were recorded as estimates of viable bacteria counts.

To assess the antimicrobial mechanisms of the TiO_2_ composite film coatings on glass fibers, the test fibers were dipped in 10^3^ CFU/mL bacterial solution. After incubation, bacteria attached to the coatings were fixed with 0.05% glutaraldehyde in phosphate buffer saline and dehydrated sequentially by water-alcohol solutions (50%, 70%, 80%, 90%, and 100% alcohol, used in this order) for 30 min in each solution. After dehydrating by a series of ethanol solutions, specimens were dried in a critical-point dryer. The samples were mounted on stubs and coated with gold. The cell wall characteristics were observed by SEM imaging.

## 3. Results and Discussion

### 3.1. XRD Results of TiO_2_ Thin Films

**Figure 1:**
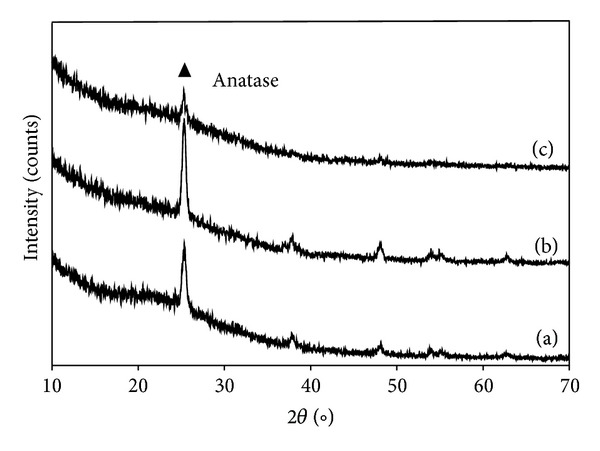
XRD patterns of TiO_2_ thin films calcined at 600°C: (a) TiO_2_, (b) 3SnO_2_/TiO_2_, and (c) 20N/3SnO_2_/TiO_2_.

Figure 1 shows the XRD patterns of the thin films, namely, undoped 3SnO_2_/TiO_2_ and 20N/3SnO_2_/TiO_2_, after calcination at 600°C for 2 h. By comparison with anatase and rutile ASTM cards (American Society for Testing and Materials, cards JCPDS 21–1272 and JCPDS 21–1276), the films included anatase phase, and the various types of thin films did not differ in these observations. During the high calcinations temperature, TiO_2_ had transformed from amorphous to anatase structure. The very broad diffraction peak at (1 0 1) plane (2*θ* = 25.3°) was due to the small crystallite size of TiO_2_. The crystallite sizes calculated from Scherrer's equation are shown in Table 1. The calcined 20N/3SnO_2_/TiO_2_ composite film had the smallest 9.8 nm crystallites. Nitrogen doping seems to hinder phase transformation from amorphous to anatase phase, leading to a low degree of crystallinity, while 3SnO_2_/TiO_2_ had the highest degree of crystallinity (Figure 1). A tetragonal Bravais lattice type was evident, and the lattice constants were calculated from diffraction peaks (*a* = *b* = 0.37821 nm and *c* = 0.95402 nm for 3SnO_2_/TiO_2_, and *a* = *b* = 0.37852 nm and *c* = 0.96917 nm for 20N/3SnO_2_/TiO_2_). Compared with anatase TiO_2_ (*a* = *b* = 0.37852 nm and *c* = 0.95083 nm), the lattice parameters *a* and *b* of 20N/3SnO_2_/TiO_2_ were almost unchanged while *c* had increased. Therefore, the doping had slightly distorted the crystal lattice structure, as expected [10]. Both crystallite size and degree of crystallinity are known to affect photocatalytic activity.

### 3.2. EDS Spectra and Morphology of Surface Thin Films

**Figure 2:**
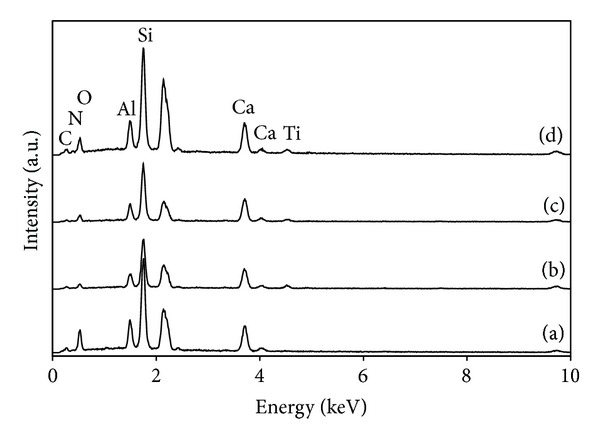
EDS spectra for (a) uncoated glass, (b) TiO_2_, (c) 3SnO_2_/TiO_2_ coating, and (d) 20N/3SnO_2_/TiO_2_ coating, all calcined at 600°C.

**Figure 3:**
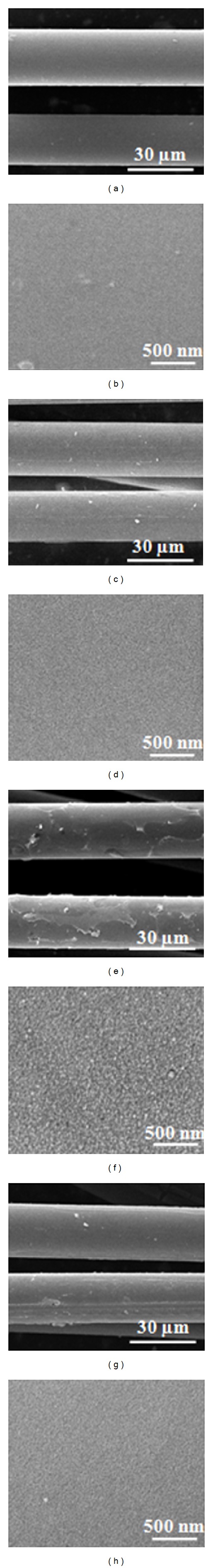
SEM images of glass fibers, some coated and calcined at 600°C: (a) uncoated 1,500x, (b) uncoated 60,000x, (c) TiO_2_ 1,500x, (d) TiO_2_ 60,000x, (e) 3SnO_2_/TiO_2_ 1,500x, (f) 3SnO_2_/TiO_2_ 60,000x, (g) 20N/3SnO_2_/TiO_2_ 1,500x, and (h) 20N/3SnO_2_/TiO_2_ 60,000x.

The EDS spectra taken from TiO_2_ and TiO_2_ composite films are presented in Figure 2. The elements Si, Al, Ca, and O were mainly in the glass fiber substrates, while Ti, N, and O elements were in the composite films from TiO_2_ and 20N/3SnO_2_/TiO_2_. The peak for Sn is not observed, presumably because of its low dosage in the composite films. The morphologies of the coated surfaces are illustrated in Figure 3, as observed by SEM. The nucleation of anatase phase was homogeneous, and the film surface was smooth. However, some excess TiO_2_ had remained randomly deposited on the coatings of glass fibers. Agglomeration of nanoparticles occurred in 3SnO_2_/TiO_2_ films, but not in 20N/3SnO_2_/TiO_2_ films. N-doping hindered anatase crystal growth, in agreement with the XRD spectra shown in Figure 1.

### 3.3. FTIR Analysis

**Figure 4:**
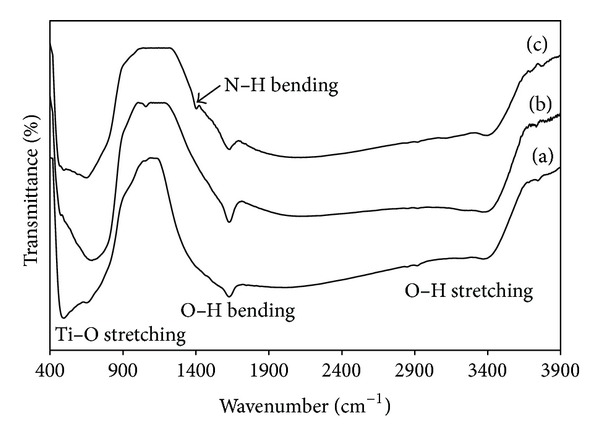
FTIR spectra of (a) pure TiO_2_, (b) 3SnO_2_/TiO_2_, and (c) 20N/3SnO_2_/TiO_2_ powders calcined at 600°C.

The photogenerated hydroxyl groups on titanium dioxide surface can be characterized using FTIR transmittance spectra especially the peaks at 3200–3600 cm^−1^ [11, 12].  Figure 4 shows the FTIR spectra of TiO_2_, 3SnO_2_/TiO_2_, and 20N/3SnO_2_/TiO_2_ calcined at 600°C. The bands appearing at about 3400–3468 cm^−1^ in TiO_2_, 3SnO_2_/TiO_2_, and 20N/3SnO_2_/TiO_2_ coatings correspond to stretching vibrations of OH groups linked with titanium atoms (Ti–OH). This confirms that photocatalytic reactions took place on the sample surfaces. The broad and strong peaks at 1630–1640 cm^−1^ are ascribed to the bending vibration of OH groups, of free or absorbed water [13, 14]. The peaks at 1403 cm^−1^ in the spectrum of the 20N/3SnO_2_/TiO_2_ sample are assigned to the vibrations of N–H bonds [15]. The peak at 600 cm^−1^ is ascribed to absorption bands of Ti–O and O–Ti–O, related to flexion vibration [16].

### 3.4. Energy Gap Measurement

**Figure 5:**
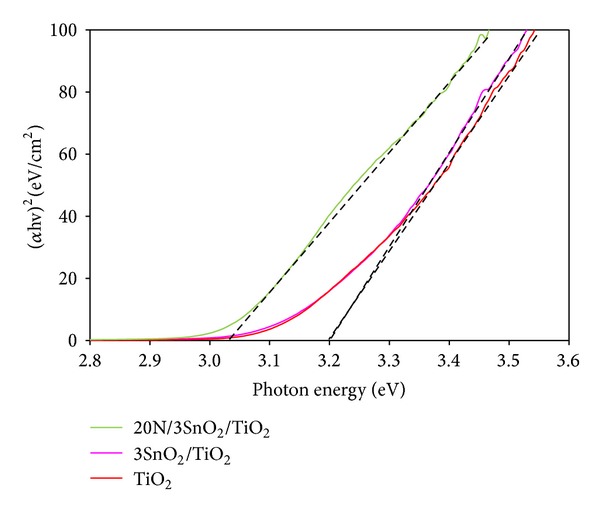
The photon energy versus (*α*hv)^2^ curve of representative pure TiO_2_, 3SnO_2_/TiO_2_, and 20N/3SnO_2_/TiO_2_ samples calcined at 600°C.

The photon energy versus curve of pure TiO_2_, 3SnO_2_/TiO_2_, and 20N/3SnO_2_/TiO_2_ are shown in Figure 5. The absorption edge energies were determined from the following relation:

(2) $$\begin{array}{c} E_{g}=\frac{1239.8}{\lambda }, \end{array}$$

where *E*_*g*_ (eV) is the band gap energy of the sample and *λ* (nm) is the onset wavelength of the spectrum. The dopants affected the UV-Vis spectra by inhibiting recombination of electron-hole pairs, especially in the case of N-doping. The band gap energy of N-doped TiO_2_ is shifted by 0.17 eV from the 3.20 eV of pure TiO_2_ (Table 1), and 3SnO_2_/TiO_2_ showed a smaller shift to 3.20 eV. These effects suggest a strategy for mediating photocatalysis through atomic-level doping of nanocatalysts. It can be seen that the absorption wavelength of 20N/3SnO_2_/TiO_2_ photocatalyst is extended towards visible light (*λ* = 409.2 nm) relative to other varyingly doped samples [16] or pure TiO_2_. The nitrogen doping hinders the growth of anatase phase (Figure 1) or it can reduce the crystallite size of TiO_2_ composite films to be about 10 nm (Table 1), leading to a quantum confinement effect of nanocrystals and the highest photocatalytic activity.

### 3.5. XPS Analysis

**Figure 6:**
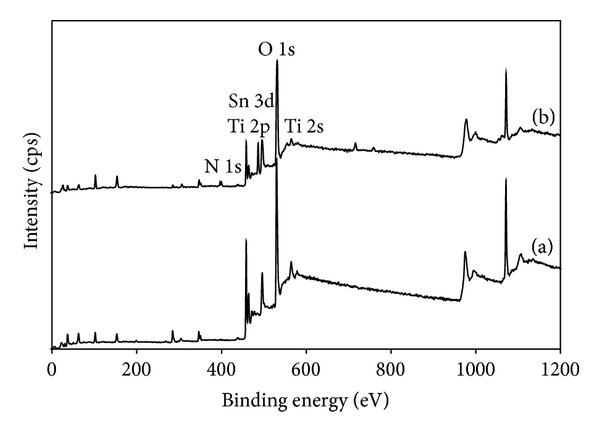
XPS spectra of (a) TiO_2_ and (b) 20N/3SnO_2_/TiO_2_ thin films, calcined at 600°C.

**Figure 7:**
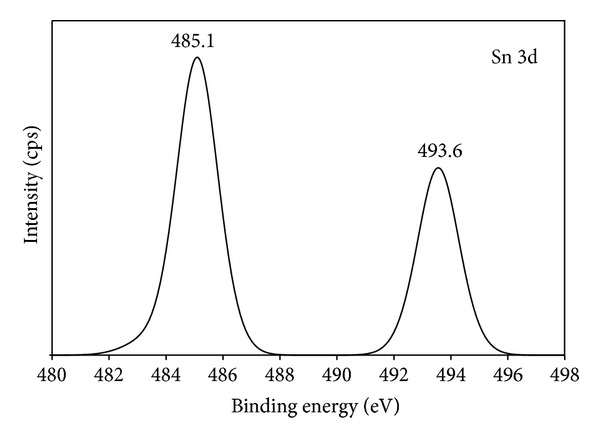
XPS spectrum of Sn 3d on the surface of 20N/3SnO_2_/TiO_2_ thin film, calcined at 600°C.

**Figure 8:**
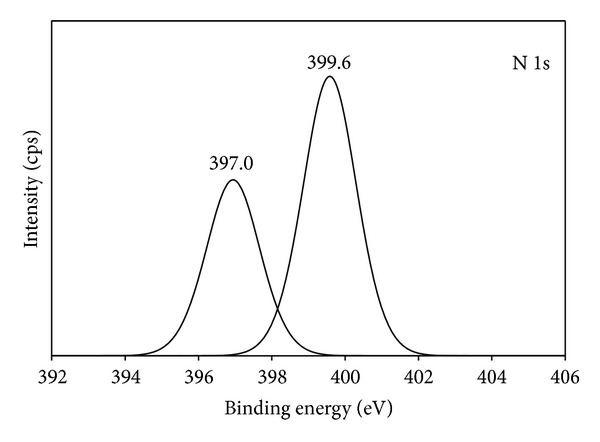
XPS spectrum of N 1s on the surface of 20N/3SnO_2_/TiO_2_ thin film, calcined at 600°C.

Figure 6 shows the X-ray photoelectron spectroscopic (XPS) spectra of TiO_2_ and 20N/3SnO_2_/TiO_2_ thin films. The Ti, O, N, and Sn elements were detected in 20N/3SnO_2_/TiO_2_ thin films, in the respective percentages 7.25, 61.44, 1.35, and 2.39. The XPS peaks indicate that the codoped TiO_2_ powders contain Ti, Sn, O, and N elements, and the binding energies of Ti 2p, Sn 3d, O 1s, and N 1s are 458, 486, 531, and 399 eV, respectively. The Sn 3d peak in the spectrum of Sn-TiO_2−*x*_, shown in Figure 7, demonstrates tin on the surface of TiO_2_. The 485.1 eV binding energy of tin in Sn-TiO_2−*x*_ is lower than the reference 486.6 eV energy reported for Sn 3d5/2-binding [17]. To assess the chemical state of N in 20N/3SnO_2_/TiO_2_ thin films, a high-resolution XPS spectrum of N 1s was measured; see Figure 8. The N 1s binding energy peaks were broad and asymmetric, demonstrating at least two chemical states of N, with binding energies 397.0 and 399.6 eV. Each of these broad peaks was decomposed to three peaks, by curve fitting, indicating two different states of N. The main peak at 399.6 eV binding energy was attributed to the N–Ti–O environment, while the peaks at 397.0 eV were assigned to the substitutional nitrogen in the Ti–N structure [18].

### 3.6. Photocatalytic Activity Test

**Figure 9:**
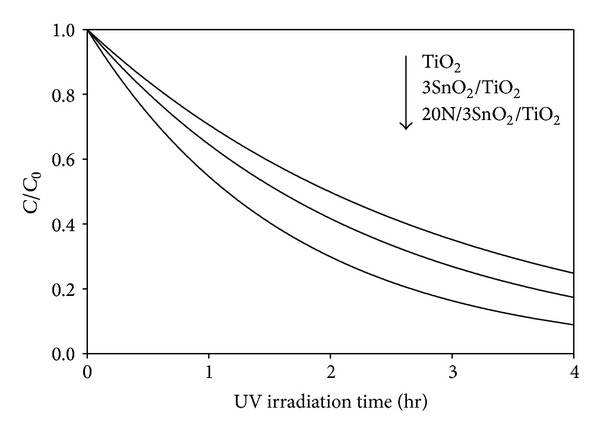
Photocatalytic degradation of MB in solution under UV excitation by various thin film coatings on glass fibers. The surface area of thin film available for reaction was held approximately constant, based on weight of glass fibers.

**Figure 10:**
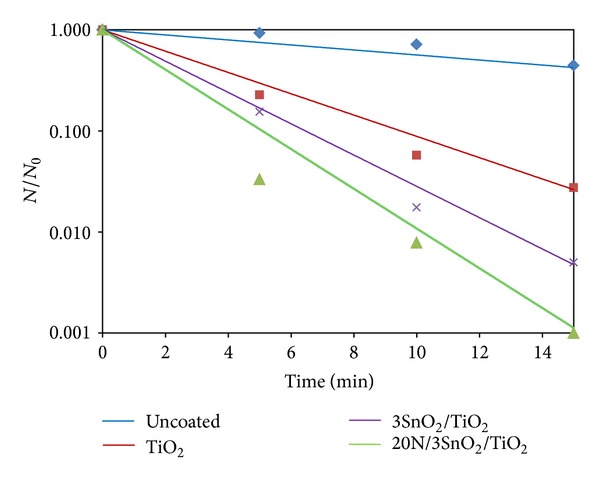
Antibacterial effects of coated glass fibers against *S. typhi* under UV irradiation.

The photocatalytic activities of TiO_2_ and the composite films were determined, by measuring degradation of methylene blue in solution (MB) with an initial concentration of 1 × 10^−5^ M, under UV for various irradiation times.  Figure 9 shows the fraction of the MB remaining versus irradiation time, which equals current concentration relative to initial concentration, *C*/*C*_0_. The 3SnO_2_/TiO_2_ had better photocatalytic activity than pure TiO_2_, possibly because photogenerated electrons can accumulate in SnO_2_ and photogenerated holes in TiO_2_, with a heterojunction formed at the SnO_2_-TiO_2_ interface. This would lower the recombination rate of photogenerated charge carriers, giving higher quantum efficiency and better photocatalytic activity [19]. The N-doped 20N/3SnO_2_/TiO_2_ thin films had the most photoactivity (Figure 10). According to prior research, factors influencing the photoactivity of TiO_2_ photocatalysts include crystalline phase, grain size, specific surface area, surface morphology, and surface state (surface OH^−^ radicals), and these factors are correlated [20, 21]. Doping TiO_2_ with N shifts its light absorption wavelength to the visible region, reduces crystallite size, and narrows its energy band gap (3.03 eV) [22]. A well-crystallized anatase phase facilitates transfer of photo-induced vacancies from bulk to surface, for degradation of organic composites, and effectively inhibits the recombination between photo-generated electrons and holes, giving excellent photocatalytic activity. As seen in Figure 1, the 20N/3SnO_2_/TiO_2_ thin film had the smallest crystallite size, estimated to be about 9.8 nm (Table 1). The reaction rate constant *k* determined is a direct quantitative indicator of photocatalytic activity (Table 2), and *k* was highest at 0.6 hr^−1^ for the 20N/3SnO_2_/TiO_2_ composite film, almost double that of pure TiO_2_.

### 3.7. Photocatalytic Disinfection against Bacteria

**Figure 11:**
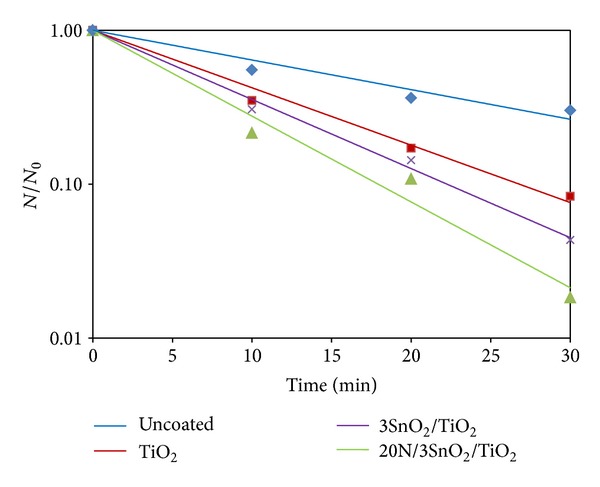
Antibacterial effects of glass fibers with various coatings against *E. coli* under UV irradiation.

**Figure 12:**
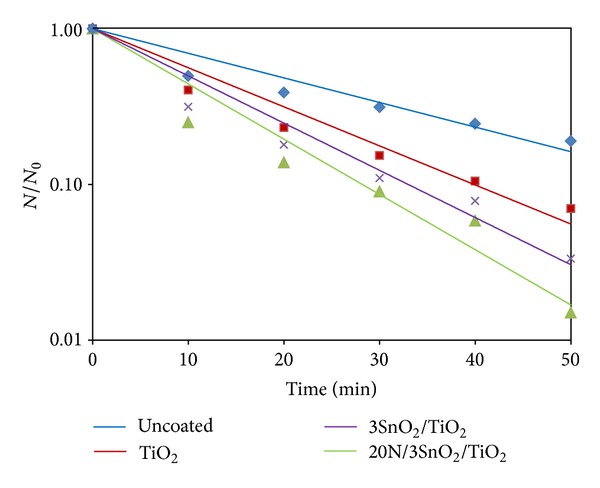
Antibacterial effects of glass fibers with various coatings against *S. aureus* under UV irradiation.

**Figure 13:**
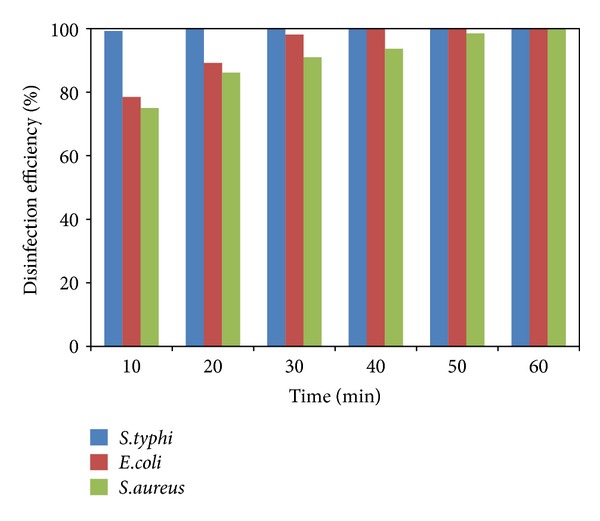
Antibacterial effects of 20N/3SnO_2_/TiO_2_ coated glass fibers against *S. typhi*, *E. coli *and,* S. aureus* under UV irradiation.

**Figure 14:**
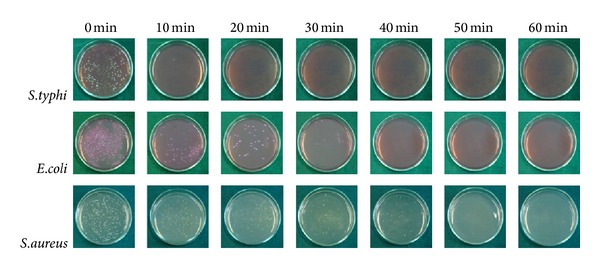
Growth of bacterial colonies on agar plates, after various treatment times. Glass fibers coated with 20N/3SnO_2_/TiO_2_ were used to treat *S. typhi, E. coli,* and *S. aureus* under UV irradiation, and the number of colonies corresponds to remaining viable count of bacteria.

**Figure 15:**
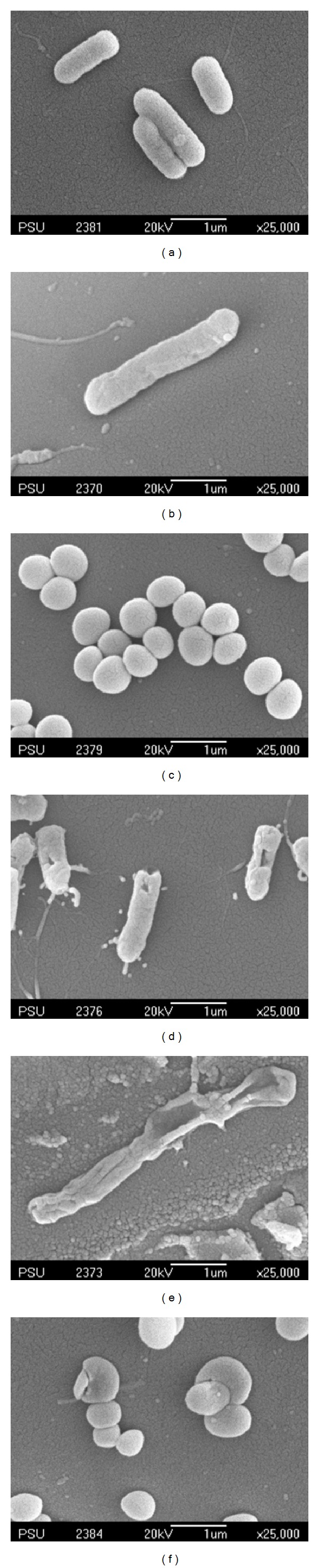
SEM images of bacteria observed on surface coated thin films: (a) untreated (*S. typhi*), (b) untreated (*E. coli*), (c) untreated (*S. aureus*), (d) irradiated for 15 min (*S. typhi*), (e) irradiated for 40 min (*E. coli*), and (f) irradiated for 60 min (*S. aureus*) of 20N/3SnO_2_/TiO_2_ composite thin films.

The photo-inactivation of bacteria, in distilled water containing the pathogen, was tested with UV excitation. The initial bacterial concentration was about 10^3^ CFU/mL, and the results are shown in Figures 10–13. The survival rates of bacteria were estimated from CFU cultures that determine the number of viable cells. The survival curves in Figures 10–12 show the fraction surviving *N*/*N*_0_, where *N*_0_ is the initial and *N* the current viable count, at a given duration of irradiation. The 20N/3SnO_2_/TiO_2_ film had the highest bactericidal activity, better than either pure TiO_2_ or 3SnO_2_/TiO_2_ with similar UV excitation, and dramatically better than UV alone. In the presence of 20N/3SnO_2_/TiO_2_, *S. typhi* was almost completely inactivated within 10 min and completely killed within 15 min. In comparison with control fibers, TiO_2_, and 3SnO_2_/TiO_2_ thin films, *S. typhi* was killed 74%, 97%, and 99.5%, respectively, after 15 min UV irradiation (Figure 10). The results shown in Figure 11 for *E. coli* are qualitatively similar, with almost complete inactivation reached within 30 min and complete kill within 40 min in the best case, and the different film types had the same rank order as above. The rank order remained the same with *S. aureus*, which was completely killed within 60 min in the best case (Figure 12). Clearly the 20N/3SnO_2_/TiO_2_ film had the best antibacterial effects against each pathogen tested. The antibacterial activity of the prepared films correlates well with the photocatalytic activity, determined from degradation of methylene blue. The inactivation rate constant, *k* of control (uncoated glass fibers under UV irradiation), TiO_2_, 3SnO_2_/TiO_2_, and 20N/3SnO_2_/TiO_2_ films determined from Figures 10–12 illustrated in Table 3, is a direct quantitative indicator of antibacterial activity. It is seen that the *k* value of 20N/3SnO_2_/TiO_2_ film was higher than that of other samples due to its smaller crystallite size or larger surface area. The killing rate, *k*, was the highest at 0.450 min^−1^ for *S. typhi* disinfection compared to 0.128 and 0.082 min^−1^ for *E. coli* and* S. aureus,* respectively.  Figure 13 shows the antibacterial efficiency of 20N/3SnO_2_/TiO_2_ composite thin film under UV irradiation. This thin film has a stronger antibacterial effect on Gram-negative than Gram-positive bacteria, because Gram-positive bacteria have thick cell walls composed of multilayered peptidoglycan [23], and also *E. coli* has thicker cell walls than *S. typhi*. The bactericidal effect of TiO_2_ has been started from the damage of bacterial outer membranes after contact with reactive oxygen species (ROS), primarily hydroxyl radicals (OH^*∙*^), which leads to phospholipid peroxidation and ultimately cell death. It has also been suggested that nanomaterials that can physically attach to a cell could be bactericidal on such contact [24]. The photos in Figure 14 show bacterial cultures on agar plates, illustrative of viable counts after various treatment times with 20N/3SnO_2_/TiO_2_ under UV irradiation. The damage to cell walls of bacteria can be immediate on irradiation in the presence of TiO_2_ thin films, and is followed by further damage to the cell membranes [25]. SEM images of bacteria before and after treatment with 20N/3SnO_2_/TiO_2_ thin films are shown in Figures 15(a), 15(b), and 15(c) and Figures 15(d), 15(e), and 15(f), respectively. The cell walls of untreated bacteria were normal, and the number of germs was high (Figures 15(a)–15(c)). After 15 min of UV irradiation the cell walls and cell membranes were damaged by contact with TiO_2_ composite films. The mechanism of this effect is that the photo-generated hydroxyl (OH^*∙*^) and super oxygen (O_2_^−∙^) radicals react, as powerful oxidizing agents, with peptidoglycan (poly-*N*-acetylglucosamine and *N*-acetylmuramic acid) of bacterialcell wall [26].

## 4. Conclusion

N-doped and undoped 3SnO_2_/TiO_2_ composite films were prepared as coatings on glass fibers, by sol-gel and dip-coating methods. The films were heated to 600°C at a rate of 10°C/min and held for 2 h, in order to form anatase phase. The 20N/3SnO_2_/TiO_2_ composite film had comparatively narrow band gap energy, high crystallinity of anatase phase, and small crystallite size as well as the highest photocatalytic activity of the films prepared. Its antibacterial activity under UV irradiation was superior to undoped TiO_2_ films, correlating well with photocatalytic activity determined from MB degradation. Antibacterial activity was experimentally established against selected bacteria of both Gram-positive and Gram-negative types, with stronger antibacterial effects against Gram-negative type. The synthesized 20N/3SnO_2_/TiO_2_ film coated on glass fibers is antibacterial photocatalyst that will be suitable for water purification.

## Figures and Tables

**Table 1:** Effect of thin film type on its anatase crystallite size, energy band gap, and photocatalytic degradation of MB in 4 h.

Samples	Crystallite size (nm)	Energy band gap (eV)	% Degradation of MB in 4 h (%)
TiO_2_	17.2	3.20	71.9
3SnO_2_/TiO_2_	17.2	3.20	80.3
20N/3SnO_2_/TiO_2_	9.8	3.03	89.5

**Table 2:** A summary of numerical fits of first-order kinetics to the degradation of MB.

Samples	Rate equation	Rate constant (*k*) (hr^−1^)	*R* ^2^
TiO_2_	*C* = *e* ^−0.34*t*^	0.34	0.952
3SnO_2_/TiO_2_	*C* = *e* ^−0.43*t*^	0.43	0.975
20N/3SnO_2_/TiO_2_	*C* = *e* ^−0.60*t*^	0.60	0.974

**Table 3:** A summary of numerical fits of first-order kinetics to the inactivation of bacteria.

Bacteria	Samples	Rate Equation	Rate constant (*k*) (min^−1^)	*R* ^2^
*S. typhi *	Uncoated	*N* = *e* ^−0.050*t*^	0.050	0.883
	TiO_2_	*N* = *e* ^−0.240*t*^	0.240	0.975
	3SnO_2_/TiO_2_	*N* = *e* ^−0.350*t*^	0.350	0.990
	20N/3SnO_2_/TiO_2_	*N* = *e* ^−0.450*t*^	0.450	0.960

*E. coli *	Uncoated	*N* = *e* ^−0.044*t*^	0.044	0.935
	TiO_2_	*N* = *e* ^−0.086*t*^	0.086	0.986
	3SnO_2_/TiO_2_	*N* = *e* ^−0.103*t*^	0.103	0.993
	20N/3SnO_2_/TiO_2_	*N* = *e* ^−0.128*t*^	0.128	0.975

*S. aureus *	Uncoated	*N* = *e* ^−0.036*t*^	0.036	0.888
	TiO_2_	*N* = *e* ^−0.058*t*^	0.058	0.940
	3SnO_2_/TiO_2_	*N* = *e* ^−0.070*t*^	0.070	0.944
	20N/3SnO_2_/TiO_2_	*N* = *e* ^−0.082*t*^	0.082	0.936
